# Identification of high risk groups with shorter survival times after onset of the main reason for suicide: findings from interviews with the bereaved in Japan

**DOI:** 10.1186/s13104-018-3672-3

**Published:** 2018-08-03

**Authors:** Kayako Sakisaka

**Affiliations:** 1 Teikyo University Graduate School of Public Health, 2-11-1, Kaga, Itabashi, Tokyo, 1738605 Japan; 2 National Center of Neurology and Psychiatry, Institute of Mental Health, Japan Support Center for Suicide Countermeasures, Tokyo, Japan

**Keywords:** Suicide, Work-related suicide, Survival times, Male, Japan, Asia

## Abstract

**Objectives:**

We sought to (1) measure survival lengths after the onset of the main reason for the target’s suicide, (2) identify the highest-risk groups and the contributors to early death, in Japan, and (3) clarify peculiar traditional Japanese values concerning suicide.

**Results:**

Data for 523 deceased individuals (median age 43.0 years) were collected from bereaved persons. Average survival time from the onset of the main reason for suicide was 1956 days (5.4 years). After controlling for confounding factors, being middle-aged, male, self-employed, and a founding company president were identified as the highest-risk groups. Half of the self-employed founding presidents died within 2 years. Many of the bereaved had observed some signs of the suicide 2 weeks ago. The traditional Japanese idea is that one means of compensating for a serious mistake is to commit suicide, and we observed that many Japanese people still regard suicide as a respectable death, even among the interviewed. The possible intervention time is quite limited; however, those who have contact with the high-risk groups should be alert to behavioral changes or signals of impending suicide.

## Introduction

### Worldwide and Japanese suicide trends

Worldwide, over 800,000 people commit suicide annually [1]. Suicide is a major public health issue worldwide [2], and the second leading cause of death among 15- to 29-year-olds [1]. Suicide account for 8.6% of deaths globally [1]. An annual global age-standardized suicide rate of 11.4/100,000 population (15.0 for males, 8.0 for females) has been reported [3]. Major risk factors for suicide are mental disorders (such as depression, personality disorder, or schizophrenia), and some physical illnesses, such as neurological disorders, cancer, and HIV infection [4]. Poverty, low income, and low social status are related to suicide rates; approximately 80% of global suicides occur in low- and middle-income countries [3]. Among high-income countries, however, Japan had the highest national suicide rate at 19.5 deaths/100,000 people in 2014, while high-income countries averaged 12.7/100,000 [5].

As indicated by a recent World Health Organization (WHO) report, one reason for the country’s continually high suicide rate is that discussing death by suicide remains a social taboo in Japan; therefore, suicide has not been addressed as a public health concern [5]. In 2006, Japan’s Basic Act for Suicide Prevention was finally signed into law [5]. Annual suicides in Japan have fallen below 30,000 from 2014; however, suicide rates, particularly among middle-aged men, were still high compared with other countries such as the United States [6]. For males in Japan, two age groups had particularly high rates: those aged 55–59 years, and 85+ years [6]. In the United States, suicide rates basically plateau in the 20–69-year age group, and then rise sharply in the 70+ age group [6]. Rates for males are much higher than those for females in both Japan and the United States [6]. Moller-Leimkuhler suggested that traditional masculinity was a key risk factor for males, and could be the major reason for the gender gap in suicides [7].

### Sharp increase in suicides among middle-aged males from 1999

In 1999, Japan saw a sharp increase in suicide rates, particularly among middle-aged males, which appeared to be attributable to economic causes [8]. Rates of work-related suicides remain high: Japanese National Police Agency statistics indicated that 9.0% (2159/24,025) of all suicides in 2015 fell in this category [9]. Work-related suicide represents one of the most challenging areas in which to implement prevention strategies [10]. WHO reports that mental disorders, harmful use of alcohol and other substances, job or financial loss, hopelessness, chronic pain, and illness are major causes of suicide [1]. Suicides that are presumably caused by work-related problems (work-related suicides) have particularly large economic impacts on the workplaces and family members of the deceased [4].

Existing studies have found that distinct life events precede suicide [11–14]; however, few studies have identified the risk factors in work-related suicides in Japan. Amagasa et al. clarified the effect of job loss on subsequent life events using work-related suicide, and outlined the process and related factors in Japan [10]. Work environments in Japan have changed dramatically since around 2000 because of economic stagnation [13]. Another study showed that factors preceding work-related suicides in Japan were low income, job loss, bullying, high demand or overwork, and low level of control in the workplace [12]. However, the interrelationships of life events and other factors in earlier suicide deaths have not been clarified.

### Objectives

We therefore sought to (1) measure survival lengths after the onset of the main reason for suicide, (2) identify the highest-risk groups and the contributors to early death in Japan, and (3) clarify peculiar traditional Japanese values, gender differences concerning suicide.

## Main text

### Methodology

#### Study design

We used a cross-sectional design. We carried out face-to-face interviews with structured questionnaires for the bereaved, who were mainly family members of suicide completers.

#### Target population

The study targeted 500 pairs of people, or 1000 individuals: 500 who died due to suicide, and one bereaved person for each deceased person. We used respondent-driven and snow-balling sampling because finding and recruiting bereaved family members of deceased persons is extremely hard in Japan because our target subjects constitute a hidden population. Our original survey, named “Listening to the Voices of the Voiceless: A Survey on Suicide” [15], was conducted by the NPO LIFELINK [16], and potential participants were identified through self-help groups for bereaved family members of suicide completers throughout Japan.

#### Data analysis

All statistical analyses were conducted by the author using SPSS version 24.0 for Windows (SPSS Inc., Chicago, IL). We set p < 0.05 to denote significant factors.

### Results

#### Characteristics of the deceased

Table 1 shows the socio-demographic characteristics of the deceased. Data for 523 individuals who committed suicide were included. Of the deceased, 69.2% were male; 30.8% were female. The median age was 43.0 [Interquartile range (IQR) 30–53] years; approximately one-third (35.5%) had graduated from high school, and another third (31.8 + 2.5%) from college/university and graduate school. The majority (62.5%) were married, and approximately one-fourth (25.4%) lived alone. Approximately 60% had no income, while nearly 10% had a monthly income of more than 400,000 yen (> 3636 USD; as of May 2018, 1 USD = 110 yen; this figure was close to the average annual income per capita in Japan) per month. Regarding employment, 41.3% were employed, 43.6% were unemployed, and 15.1% were self-employed at the time of death.

**Table 1:** Sociodemographic characteristics of the deceased

Studied variables	n	%
Sex (n = 523)		
Male	362	69.2
Female	161	30.8
Age in years (n = 523)	Median 43.0 [IQR: 30–53]	
< 20	30	5.7
20–29	96	18.4
30–39	56	19.9
40–49	104	23.1
50–59	121	20.7
60–69	108	26.0
70–79	49	9.4
≥ 80	15	2.8
Highest education level (n = 494)		
Middle school	58	11.3
High school	183	35.5
Technical college	46	8.9
Junior college	17	3.3
University	164	31.8
Graduate school	13	2.5
Others (e.g., elementary school)	13	2.5
Marital status		
Married	327	62.5
Not married	196	37.5
Number of cohabitants: Median 2.0 [IQR: 1.0–3.0] (n = 445)		
Living alone	113	25.4
2	128	28.7
3	113	25.4
4+	91	20.5
Had a job before the death (n = 522)		
Yes	312	59.7
No	210	40.3
Monthly income (USD)^a^ (n = 516)		
None	292	56.6
< 200 thousand yen (< 1739 USD)	100	19.4
200,000–400,000 yen (1739–3478 USD)	82	15.9
> 400,000 yen (> 3,478 USD)	42	8.1
Job type (n = 397)		
Self-employed	60	15.1
Employed	164	41.3
Unemployed	173	43.6
Position in the workplace (n = 326)		
Manager class or higher	120	36.8
Not manager class	206	63.2
Bank deposit, savings^a^ (n = 515)		
None	321	62.3
< 1 million yen (< 8695 USD)	80	15.5
1 million–10 million yen (8695–86,957 USD)	68	13.2
> 10 million yen (> 86,957 USD)	46	8.9
Experience of suicide attempt (n = 473)		
Yes	171	36.2
No	302	63.8
Experience of being abused (n = 398)		
Yes	76	19.1
No	322	81.9
Suicide of a family member or other relative (n = 437)		
Yes	131	25.1
No	390	74.9

^a^1 USD = 115 yen (as of June, 2018)

#### Personal life, suicidal behavior, and help-seeking behavior

Table 2 outlines personal life factors, suicide-related information, and help-seeking information of the deceased. Suicide methods varied. The majority (61.0%) of the deceased used hanging; the next most frequent category was jumping from a high place (14.7%). Nearly half (43.5%) left a suicide note.

**Table 2:** Suicide, personal life, and suicidal behaviors of the deceased

Study variables	n	%
Suicide methods (n = 462)		
Hanging	282	61.0
Jumping from a high place	68	14.7
Poisoning	29	6.3
Briquette coal	27	5.9
Brodie to train	23	5.0
Carbon monoxide poisoning	20	4.3
Burning	13	2.8
Suicide note (n = 490)		
Yes	213	43.5
No	277	56.5
Held private life insurance (n = 454)		
Yes	304	67.0
No	150	55.0
Sudden decrease in income		
Yes	61	22.6
No	209	77.4
Multiple debts		
Yes	82	15.7
No	441	84.3
Loss of employment (including bankruptcy)		
Yes	57	10.9
No	466	89.1
Deteriorated relationships in workplace		
Yes	95	18.2
No	428	81.8
Sleep disorder observed		
Yes	250	73.3
No	91	26.7
Change of daily life behaviors observed		
Yes	290	69.2
No	24.7	30.8
Attended psychiatric consultation (medical treatment)		
Yes	241	46.1
No	282	53.9
Consulted with friends, colleagues		
Yes	415	79.3
No	108	20.7
Suicidal behavior before suicide (n = 430)		
Yes	352	81.9
No	78	18.1
Timing of recognized suicidal behavior (n = 158)		
Less than 7 days before the death	35	22.2
7–14 days before the death	46	29.1
15–30 days before the death	9	5.7
More than 30 days before the death	68	43.0
Recognized signs and symptom of suicide (n = 467)		
Yes	298	63.8
No	169	36.2
Timing of signs and symptoms of suicide (n = 132)		
Within 14 days	75	56.8
14–30 days	21	15.9
More than 30 days	36	27.3

More than two-thirds (67.0%) had private life insurance, which normally can be paid out for suicide deaths in Japan [17, 18]. Half of the interviewees (22.2 + 29.1%) observed unusual behaviors such as cleaning desks, visiting relatives, or contacting old friends within 2 weeks before the suicide.

#### Median survival days from onset of main reason for suicide, and associated factors

Table 3 shows survival days from the onset of the main reason for suicide to death, and possible associated factors (Mann–Whitney U test). Median survival time was 1956.0 days (5.5 years) [IQR: 658.5–4035.5 days]. Personal risk factors for shorter survival time included being male (median 1430.0 days = 3.9 years, p < 0.001), under 20 years old (median 1199.0 days = 3.3 years), 20–29 years old (median 1664.0 days = 4.6 years), or 40–49 years old (median 1778.5 days = 4.9 years).

**Table 3:** Days from onset of main cause of suicide to suicide completion, and determinants of early suicide death (< 3 years from onset of main cause to suicide)

Variables	n	Median		IQR^a^ (days)	p-value	
		(Days)	(Years)			
Total	397	1956.0	(5.4)	658.5–4035.5		
Male	265	1430.0	(3.9)	478.0–2928.0	< 0.001***	
Female	132	3185.0	(8.7)	1452.0–5825.5		
Age	(n = 396)					
< 20	21	1199.0	(3.3)	444.0–2507.0	0.014*	
20–29	82	1664.5	(4.6)	516.3–3124.3		
30–39	76	2290.0	(6.3)	744.0–3937.5		
40–49	86	1778.5	(4.9)	717.0–3947.0		
50–59	79	2350.0	(6.4)	749.0–4591.0		
≥ 60	52	2494.0	(6.8)	496.8–6185.8		
Job type	(n = 397)					
Self-employment, employer	60	828.0	(2.3)	388.3–2616.5	0.001**	
Employee	164	1813.0	(5.0)	772.3–3557.3		
No job	173	2533.0	(6.9)	935.5–5114.5		
Self-employed president	(n = 67)					
Founder	41	697.5	(1.9)	365.3–2863.0	0.004**	
Successor	26	2173.0	(6.0)	710.0–5567.0		
Private life insurance	(n = 356)					
Yes	228	1628.5	(4.5)	585.0–3573.3	0.043*	
No	128	2350.0	(6.4)	901.5–4442.3		
Slump in business	(n = 397)					
Yes	44	1106.0	(3.0)	608.8–2479.5	0.029*	
No	353	2059.0	(5.6)	692.5–4245.0		
Working status	(n = 249)					
Managing director or higher	104	1262.0	(3.5)	372.3–2571.5	0.001**	
Not managerial post	145	2290.0	(6.3)	736.0–5005.5		
Job Promotion	(n = 397)					
Yes	15	1219.0	(3.3)	163.0–1956.0	0.036*	
No	382	2018.0	(5.5)	701.8–4171.8		
Excessive fatigue (overwork)	(n = 397)					
Yes	54	1291.0	(3.5)	343.0–2010.3	< 0.001***	
No	343	2150.0	(5.9)	767.0–4323.0		
Experience of abuse in the past	(n = 373)					
Yes	73	3113.0	(8.5)	1529.5–5501.0	< 0.001***	
No	300	1658.5	(4.5)	573.5–3522.0		
Experience of suicide attempt	(n = 373)					
Yes	146	2603.0	(7.1)	393.0–3358.0	< 0.001**	
No	227	1464.0	(4.0)	1310.3–4947.5		

Studied variables	Crude odds ratio	95% CI	p-value	Adjusted odds ratio	95% CI	p-value
Determinants of early death (< 3 years) from the onset of main cause of suicide (logistic regression analysis)						
Self-employed, founder president	4.82	2.39–9.74	< 0.001***	4.52	2.01–10.15	< 0.001***
Had a job, employee	1.61	1.05–2.47	0.030*	3.93	1.62–9.54	0.002**
Managerial position	1.65	0.98–2.78	0.058			
Male	2.85	1.73–4.70	< 0.001***	2.51	1.02–6.20	0.046*
Age quartile	0.99	0.98–1.01	0.262			
Experience of abuse	0.31	0.16–0.62	0.001**			
Experience of suicide attempt	0.37	0.23–0.60	< 0.001***	0.35	0.17–0.69	0.003**
Family member’s with suicide history	0.77	0.47–1.25	0.288			
Consulted with someone	0.48	0.29–0.80	0.005**	0.53	0.30–0.95	0.033*

Backward elimination was used to generate the best model

We entered variables with p < 0.05 in binary regression as covariates for the final model

* p < 0.05, ** p < 0.01, *** p < 0.001

^a^ Internal quartile range

We ran binary and multiple logistic regression analyses to determine higher risk factors of early death, defined as less than 3 years from the onset of the main reason for suicide (Table 3). We mainly analyzed variables with p < 0.05 in the Mann–Whitney U test in the regression analyses. After controlling for possible confounding factors, we ran multiple regression analysis. We adopted backward elimination to generate the best model. In the final model, we identified the following positively associated high-risk factors: self-employed founder-president status [Adjusted odds ratio (AOR) 4.52, 95% CI 2.01–10.15, p < 0.001], employee status [AOR 3.93, 95% CI 1.62–9.54, p = 0.002], and being male [AOR 2.51, 95% CI 1.02–6.20, p = 0.046]. In contrast, previous suicide attempts [AOR 0.35, 95% CI 0.17–0.69, p = 0.003] and consulting with anyone in advance [AOR 0.53, 95% CI 0.30–0.93, p = 0.033] were associated with more survival days.

#### Main findings

Our study identified that median survival length from the onset of the main reason for suicide among the deceased was 5.4 years (median 1956 days). The highest-risk group (fewest survival days) were male self-employed founder-presidents. Half of the founder-presidents died within 2 years of onset of the precipitating cause (median 1.9 years). Similarly, employees in director or manager positions, and those who received a job promotion were also at risk of significantly fewer survival days. The majority of the deceased, however, showed several suicidal behaviors or other signals before the suicide. Almost half of respondents noticed such occurrences approximately 1 month (30 days) before suicide. Surprisingly, a majority (79.3%) of the deceased sought help from someone, while those who had shorter survival times tended not to do so.

### Discussion

#### Possible reasons for associations, and comparison with other studies

Our high-risk group had work-related precipitating causes, although the most frequent reasons for suicide in the national data are health-related problems [19]. Among the deceased, for those who were self-employed at the time of death, such as founder-presidents whose data are shown in Table 3, financial problems seemed to be the strongest reason that eventually led them to kill themselves after a shorter period, which supports a previous finding [20]. Regarding the unemployed, health problems accounted for the greatest number of cases.

This study might suggest that the deceased self-employed tried to compensate for their debt with private life insurance payments to protect their bereaved families, since 67% had life insurance. In Japan, life insurance companies can pay death benefits even in the case of suicide, if the 2- to 5-year exemption period has passed [17, 18]. These death benefits might constitute a moral hazard for increasing suicide deaths.

The path to suicide for the self-employed could begin with business failure, which existing evidence shows may lead to hardships in life, multiple debts, or overwork, followed by depression [3, 10]. For the unemployed, the path may begin with absence of a job or job loss, or some individuals may face workplace difficulties, such as heavier workloads or longer working hours, which can lead to health problems, eventually causing them to relinquish their work [2, 10, 20].

This study included those who presented risk factors for suicide, such as mental health problems including depression [21], and a family history involving suicide/attempts or personality disorders [22]. Although the risk factors themselves indicated suicidality, it is difficult for family members and surrounding people to recognize all mental health problems. Regarding work-related suicide, interpersonal conflicts were common among the deceased; therefore, preparing people to cope with such conflicts [23] and increasing the availability of relationship counselling are necessary [24].

#### Peculiar traditional Japanese values concerning suicide

Worldwide, poverty and low social status are associated with high suicide rates [3]. Our study, however, showed a positive association between fewer survival days and higher social status, such as for company owners, managers, and those who have been promoted (Table 3). The context of these associations might be the failure of company management or workplace difficulties [10, 20, 25, 26]. The traditional Japanese idea is that one means of compensating for a serious mistake is to commit suicide, and many Japanese people regard suicide as a respectable death [27].

#### Gender differences

Surprisingly, this study identified a negative association with previous suicide attempts. Existing study emphasized that a suicide attempt is the strongest risk factor for committing suicide [3]. The present study, however, demonstrated that experiencing abuse or suicide attempts might contribute to longer survival times after the onset of the main reason for suicide. Perhaps abuse—for example, child abuse, domestic violence experiences—includes “family problems” that occur repeatedly over a long period. These factors were more likely to be observed in females than males [28, 29]. In contrast, debt and company mismanagement related to short-term deadlines for payment, accrual of interest, or imminent judgments of bankruptcy may have affected males [3, 10, 28].

#### Recommendations

We observed that the possible intervention time is quite limited; however, those who have contact with high-risk groups should be alert to behavioral changes or signals of impending suicide.

## Limitations

To the best of our knowledge, this is the first study to estimate the average survival days after the onset of the main reason for suicide, and was based on interviews with more than 500 bereaved persons in Japan.

This study, however, has several limitations. First, sampling was respondent-driven and used snow-balling because families of those who commit suicide are reluctant to speak out. Although the interviewees gave us much useful information, we must consider the possibility of response bias in their answers.

## Abbreviations

AOR: adjust odds ratio
COR: crude odds ratio
HIV: human immunodeficiency virus
IQR: interquartile range
NPO: Nonprofit Organization
WHO: World Health Organization
